# When things go wrong: experiences of vaginal mesh complications

**DOI:** 10.1007/s00192-022-05422-z

**Published:** 2023-01-06

**Authors:** Bridget Dibb, Fee Woodgate, Lauren Taylor

**Affiliations:** School of Psychology, University of Surrey, Guildford, Surrey GU2 7XH UK

**Keywords:** Vaginal mesh implants, Attitudes of the medical profession, Loss of sexual intimacy, Pain, Personal growth, Support, Qualitative

## Abstract

**Introduction:**

Previous research has suggested that complications stemming from vaginal mesh can lead to life-changing negative physical consequences including erosion and chronic pain. However, there has been little research on the experiences of women who have had complications. This study was aimed at exploring the individual experiences of women who have had vaginal mesh complications and how this has impacted them.

**Methods:**

An explorative qualitative design was followed. Eighteen semi-structured interviews were conducted with women who had experienced complications with vaginal mesh due to stress urinary incontinence and pelvic organ prolapse. The mean age was 52 and the mean time since the mesh was fitted was 8 years (6 had since had it removed and a further 6 had had partial removal), and the mean time since first mesh-related symptom was 10 months. Data were analysed using thematic analysis.

**Results:**

Four main themes were identified: perceived impact of mesh complications, attitudes of medical professionals, social support and positive growth. Results showed that participant experiences of their mesh complication were psychologically traumatic, including feelings of increased anxiety and fears relating to suicidal thoughts. Intimate relationships were also affected, with reduced sexual functioning and intimacy stemming from mesh complications. Negative experiences with medical professionals included feeling dismissed, a lack of recognition of their symptoms, and anger towards the profession.

**Conclusions:**

The impacts of vaginal mesh complications were found to be wide-reaching and life-changing, affecting numerous aspects of participants’ lives. Greater awareness in this area is needed to provide further support for women experiencing vaginal mesh complications.

## Introduction

Synthetic mesh is a common method of treating stress urinary incontinence (SUI) and pelvic organ prolapse (POP), both conditions that are common after childbirth [1, 2]. The treatment involves implanting the mesh so that it forms a sling to support the bladder and relieve symptoms. Although the majority of women report treatment success [3] and satisfaction [4], a growing number of women are reporting complications [5]. In the period between 2005 and 2010, a total of 3,979 complications with vaginal mesh were reported, including malfunctions, injury and death. Of those, 2,874 had been filed in the last 3 years of data collection (2005–2010), alluding to the rapid increase in the complication rate.

Reports show that, 10 years after the vaginal mesh was fitted, health outcomes were less favourable than previously reported [6, 7]. These findings may be more serious than apparent as these complications are often underreported [8, 9], and underappreciated by both medical professionals and patients with mesh [8]. It has been suggested that 1 in every 30 women may need a second procedure to remove or revise the mesh, up to 10 years after surgery [10], with some requiring multiple interventions [11]. Further surgeries can result in residual symptoms [12] and emotional distress [1].

Women’s lives have been irreversibly altered for the worse owing to mesh complications, causing chronic pain and impacting on their quality of life [7, 8, 13]. Physical pain includes pain during sexual intercourse (dyspareunia) [13]; pelvic pain [11, 14]; mesh degradation [15]; and mesh exposure (erosion and extrusions) [16]. Research into the psychological impact is sparse; however, low quality of life ratings have been reported in some studies [1, 7, 17, 18]. Although this research suggests some understanding of the possible difficulties after insertion of vaginal mesh, further research showing the individual’s experiences of these complications will give more depth and understanding. Thus, this study was aimed at further exploring the experiences of women with vaginal mesh complications through interviews to gain a subjective understanding of their experiences.

## Materials and methods

### Design

The study followed a qualitative design to allow an in-depth exploration of the experience of complications with vaginal mesh implants.

### Participants

Women (*n* = 18) were residents in the Southeast of England, described their gender as female, and had a mean age of 52 (range 40–67). Mean time since the mesh was fitted was 8 years (range = 4–15 years). Mean time since the first mesh complication symptom was 10 months (ranged from straight after the operation to 4 years post=operation). Table 1 presents background details of the need for the implant (SUI and POP), type of implants, and the number who had their mesh removed.

**Table 1:** Descriptive data

	*N* (%)
Presenting condition	
Stress urinary incontinence	14 (78)
Pelvic organ prolapse	2 (11)
Both	2 (11)
Type of mesh implant	
Transobturator tape	3 (17)
Tension-free vaginal tape	7 (39)
Tension-free vaginal tape-obturator	3 (17)
Not specified	5 (28)
Mesh removal	
Complete removal	6 (33)
Partial removal	6 (33)

### Procedure

Ethical approval was received from University of Surrey Ethics Committee. A purposive recruitment strategy was used to recruit 18 participants, all via a social media support group. Interested participants were interviewed face-to-face and consent was gained at the time of the interview. The interview schedule (piloted on one participant) included broad, open questions to encourage the participants to discuss their personal experiences. Questions focused on the experiences of vaginal mesh, the response from health care professionals, what was helpful during their experiences and whether there were any positive aspects of their experience. Participation was confidential, with all participants assigned a pseudonym.

### Analysis

The transcripts were analysed using thematic analysis, following Braun and Clarke’s framework [19]. This involved initial coding of meaning (including both semantic and latent codes); codes were sorted into initial themes and then clustered into final themes and subthemes. To reduce bias, a reflexive approach was followed by the authors (FW and BD) in the interview schedule design and theme development.

## Results

Participants all experienced complications from a mesh implant and all describe in detail their perception of what went wrong, for example, Marie said “finally I discovered what was wrong with me, like basically it was cutting, it was sort of embedding itself into my urethra so it would, she [Health Professional] reckons that in another maybe few weeks it would have cut through so she had to do a urethroplasty where she reconstructed the urethra”. The analysis and theme development focused on these experiences rather than on medical complications themselves. Table 2 displays the four main themes identified following data analysis. Theme 1, perceived impact of mesh complications includes subthemes: negative emotional impact, and loss of sexual intimacy. Theme 2, attitudes of medical professionals, shows how participants viewed the interactions with the medical profession. Theme 3, social support, focuses on the support participants received from partners and friends. The final theme, positive growth shows how participants perceived some growth from their experiences.

**Table 2:** Themes and subthemes

Theme	
1. Perceived impact of mesh complications	
1.1 Negative emotional impact	
1.2. Loss of sexual intimacy	
2. Attitudes of medical professionals	
3. Social support	
4. Positive growth	

### Theme 1: perceived impact of mesh complications

The participants described how they felt about their complications with their mesh implants. They reported a range of negative emotions and described the negative impact on their intimate and sexual relationships (loss of sexual intimacy).

#### Negative emotional impact

Most participants experienced negative emotions as a result of mesh complications and subsequent continuous pain. These ranged from feeling robbed; trapped; regretful; anxious, and suicidal. Below, Frida describes feeling let down and punished,

So yeah, I just feel so let down. I feel like, and I’ve been here and I’ve been butchered, I’ve been [sigh], I don’t know, I just feel I’ve been punished. (Frida)

Participants also described feeling that their lives had had a dramatic change and that they now felt “robbed” of the life they had before,

So you do feel like you’ve been robbed of your, your life you know, it’s not the same, it’s not the same life I had before, like you know, completely different. (Liz)

Anger towards medical professionals was also experienced by others who felt that an injustice had been done to them,

All I wanna do is go how angry I am and how I want to kill the surgeon who put it in in the first place, and for lying to me, and get him struck off and ruin his life. (Diana)

The anger, though often about the injustice of what had happened, was also expressed in terms of self-blame,

It’s made me quite angry, but, I’m also angry at myself, ‘cause I, I wished I’d looked into it, I just thought it was gonna be a quick fix, I really did. (Helen)

Anxiety was also reported by participants, not just as a result of the trauma from the complications but also because of concerns over being dismissed by the medical profession,

I’ve felt [my] heart flutter when I went to see the GP, fear, you know, of being dismissed again of being sort of dismissed again as somebody who can’t cope. (Anne)

Several participants reported feeling suicidal and one participant was admitted to hospital after taking an overdose. There was a sense that being in constant pain and feeling unable to cope became unbearable for these women,

At the times when I was suicidal it was … it was just horrendous, you know, because … I would sit here, crying, you know with pain, thinking there isn’t any point in carrying on at all, you know because … there was just nothing I could do without pain, you know. (Theresa)

And,

I did have suicidal thoughts when I thought nobody believed me. I thought I was going mad, because that’s what they made me feel, and I was still in pain, and I’d had another night where I’d had no sleep, and my husband had popped out, I just looked at all my painkillers and I thought you know what, I’m just gonna take them all. That’s how low, that was my lowest point. (Liz)

Bea and Theresa, below, show how they felt that they were now changed, they felt “old” and “like a shadow”, highlighting how these changes were hugely impactful in their lives,

Sometimes when I’m out I feel like I’m a bit of a shadow that don’t wanna be seen … yeah I feel like I wanna shuffle around and be invisible, you know please don’t notice me, I just wanna go in the shop and do what I’ve got to do, if I can remember … and then go home again … that has changed massively. (Bea)

And,

I felt like an old lady, you know, I was walking with my head down and shuffling along because everything, because it’s here, everything sort of radiates out. (Theresa)

Participants also expressed regret that they had undergone the procedure in the first place, with a preference for incontinence rather than experiencing a life with complications,

But oh god, how I wish I hadn’t had that done, in fact I probably wouldn’t have bothered, I would have just put up with wearing Tena ladies. (Julie)

#### Loss of sexual intimacy

The experience of pain was wide-reaching for these women, extending beyond their personal experiences and into their intimate relationships. Comments show how painful sex had become:

Me and my husband didn’t have sex for a year, and then I just have to go, well I’m going to yell in pain, but you know, if you want to it’s fine, but you know, you can’t have that relationship with someone screaming in pain going ow ow ow, can you? (Diana)

Some women were also left feeling unattractive as a result of not being able to have sex and the stress that this put on their relationship,

I think, the inability to … to have a sexual relationship you know, sort of is another sort of very, difficult journey you know, to deal with I think, because you feel unattractive you know, you feel … that there is a strain on the relationship because of it. (Anne)

The lack of sexual relations was linked to feelings of loss and the relationship becoming more of a friendship,

For me, the closeness with him has gone we’re more like mates, more like roomies […] It’s completely taken that that closeness, the intimacy’s gone, completely gone and I don’t think that’ll come back now ‘cause it just feels so awkward. (Bea)

### Theme 2: Attitudes of medical professionals

Many participants trusted their medical professionals throughout their experiences, particularly when they were initially deciding whether to have mesh fitted. Participants recalled how they were told that the mesh was the “best thing since sliced bread” and a “quick fix”. Participants trusted their doctor because they assumed that the doctor knew best. The participants felt that medical professionals appeared to paint a simplistic picture of a quick and pain-free operation that would allow them to return to the normalcy of continence that they yearned for. Their trust in physicians enabled this process to happen with few questions asked,

He told me about the mesh and how it would revolutionise my life, um you know quick 20-minute operation, day case operation, you won’t even have any pain, nothing, you’ll be fine um and you’ll be able to run, laugh, jump, do what you like. So, I said fantastic, brilliant, that’s just exactly what I need. (Marie)

However, participants felt that the attitudes of the medical profession were dismissive when it came to the concerns and worries that they had about the pain they experienced after the implant. Some women reported that their doctors normalised the pain that they were experiencing and dismissed their symptoms, as the initial problem, the incontinence, had been remedied by the operation,

When I had the check up after he said oh you’ll get used to the pain … are you still incontinent? And I said no, so he said well I won’t be reporting it then because the operation’s worked as far as I’m concerned. (Theresa)

And

Um but every time I’d go back into hospital I’d say things like, do you not think it could be this mesh, I’d be told oh no, don’t worry it can’t possibly be your mesh. (Liz)

Below, Anne shows how her inability to empty her bladder was dismissed by her consultant and she even felt guilty for troubling him,

I was having a lot of pain so um … rather insensitively all I got through from my consultant was … a leaflet saying this is how you self-catharise please make an appointment with a nurse … eventually I got an appointment with him and he, er said well there’s nothing I can do it’s a permanent thing now and you know you your incontinence is solved isn’t it? So, um, you know, he dismissed my my problems of pain, he dismissed my … problems of you know not being able to empty properly so … I was being made to feel guilty that I actually bothered him you know because he had solved the problem … as far as he was concerned. (Anne)

Participants reported that the medical professional responded to their further queries in a patronising way and that they were viewed as “hysterical”,

When they first found the mesh had gone through and said, you know, but don’t worry, there’s a bunch of hysterical women on the internet, like you know, just close your ears to them like, you know, that kind of attitude. (Liz)

and,

But then you get that derogatory “oh you’ve been on the internet”, no they, they, patronise and I think they make you feel so worthless, you know. (Helen)

Participants felt that their worries and concerns about new and ongoing pain were dismissed as old age, or normal post-operative pain, and not taken seriously,

My hip started really hurting on the side and … I had to stop and just sit on the wall for a little while and I thought blimey I’m getting really old, I mean even old grannies are going past me and I thought this can’t be right, so I went back over and they said right we’ll X-ray … and they said it’s um … mild degenerative arthritis so basically old age. (Anne)

And,

He’s very much like ah you’re fine, you’re fine, it’s your menopause, it’s your menopause, you know everything’s the menopause or fibromyalgia. (Bea)

### Theme 3: Social support

Participants spoke warmly of the support they received from others: from friends, partners and support groups,

I think the support of some friends who you know did … rally round then and sort of, you know, drove … drove me, you know, in or, you know, supported me. (Anne)

And,

Finding [a support group] and finding there’s other women out there, that I wasn’t alone, um just made me feel, not that I wanted other women to have it, but it kind of makes you feel not so alone, you can all understand what each other’s going through. So the campaign on Facebook’s been, I mean, it’s been wonderful. (Liz)

Further reports of positive support came from finding relief in knowing others with similar experiences. This served to validate their experiences, reassuring them that it was not just heir fabrication, as some medical professionals had made them feel,

Just having someone go, yes actually, there’s probably a physical cause for your, for these years, […] and the relief, of not, and that’s awful as well, the relief of it not being in your head, and being able to say to people, actually it’s not in my head. (Beatrix)

Participants often commented on support from their partners. Below Emily speaks of the help her husband provided,

When I’ve got to the point where I just don’t know what to do, he just finds some way of doing it, and he’d write a letter or, you know, just, you know, not pushing you into anything. (Emily)

However, although social relationships tended to be framed positively, some participants spoke of times when it could feel negative,

One day I actually asked him [husband], um, I said you know what I need, you know what my problem is, and he said I’m sorry I can’t help you, it’s your issue, you need to deal with it. So I was shattered by that comment, because I thought … you could have even met me half way. (Mary)

### Theme 4: Positive growth

Despite the clearly negative experience of the mesh complications, many participants spoke of positive personal growth as a result of the experience. Some now felt able to question advice given to them, others found new opportunities to give back by campaigning for change. Bea described how she had gained more agency in decision making,

What I’ve learnt from it, is not to just sit back and just accept um what professionals say. I have a bit of a mistrust of them anyway … um … but now, I won’t sit there and be embarrassed, I will sit there and say well mistakes have been made in the past, and I’m one of them. So I can ask you a question if I choose, I don’t need to take your opinion just as like truth so it’s really taught me a lot about standing up for myself. (Bea)

Some felt empowered,

I’m strong anyway but it’s given me the … it’s given me the power to open my mouth and say, actually I’m not right, this needs to be sorted. (Marie)

And some participants demonstrated resilience in the form of acceptance,

I just tend to accept it I suppose, really, that’s the way things are, there’s nothing I can do to change what’s happened. But, I might be able to change what happens in the future (Meryl)

Others found positives in being able to help others, both in terms of campaigning for the rights of women who had experienced mesh complications and offering advice and support to others in similar situations,

I still feel a lot of anger, and then channelling it into campaigning has been the most positive thing. (Diana)

## Discussion

This study was aimed at exploring the experiences of women with vaginal mesh complications, an understudied topic. Previous research in this area has predominantly been quantitative and focused on the medical impacts of vaginal mesh [11, 13, 16]. This research raises awareness of the experiences of these women and the support they need. Themes including the emotional impact, interactions with medical professionals, the support they received, and personal growth, were developed. Participants gave vivid descriptions of the physical damage and the resultant pain caused by mesh implants, highlighting the severity and wide-reaching impacts of their experiences. These results are supported by a recent focus group study illustrating the trauma participants experienced, how it impacted hugely on multiple domains of their lives, interfering with intimate relationships and day-to-day living [7].

These results build on Dunn et al.’s [1] qualitative study, which mirrors much of the above findings, with many participants feeling anxious and desperate. Negative emotions have been shown to correlate negatively with quality of life [20, 21] and indeed, these participants reported feeling like a “shadow” of their former selves. A number of participants reported feeling suicidal, “I did have suicidal thoughts … I just looked at all my painkillers and I thought … I’m just gonna take them all… that was my lowest point” supporting the reports of distress in other studies. “I can’t even think about it … because that’s when the depression kicks in” [1]. This is especially poignant as the risk of suicide is double in patients with chronic pain [22], highlighting the importance of raising awareness of the experiences of mesh complications. Although previous research has highlighted the impact on sexual activity and resulting dyspareunia [1], this study details how mesh complications impacted not just sexual activity but also the quality of their relationship, particularly in terms of the loss of intimacy within the relationship.

The second theme, attitudes of medical professionals, showed a predominantly negative picture of the medical support received by these participants once the complications had arisen, including perceptions of dismissiveness. This supports Uberoi et al.’s [7] study, where participants felt dismissed and Dunn et al.’s [1] study, which also found difficult relationships with physicians as a result of participants’ mesh experiences. Similarly, a study on breast implants showed that the women felt disregarded by doctors [23]. These negative feelings are concerning as they may lead to reduced help seeking, which has been found in patients with cancer, where a lack of confidence in the health care profession was given as a reason for not seeking help [24, 25].

The third theme, social support, showed how participants found the care from friends, partners and support groups to be beneficial. This corroborates the vast social support literature showing the positive role of social support in living with chronic conditions [26, 27]. This theme illuminates the importance of including social support in any future interventions aimed at helping women with mesh implant complications.

The final theme focused on reports of positives within the participants' experiences. This showed how personal growth inspired confidence and was associated with greater acceptance of their situation. Post-traumatic growth is associated with psychological adjustment and positive health outcomes in other conditions, such as cancer [28] and HIV [29]. The presence of positive growth in women with mesh complications is also testament to the gravity of their experiences, as this is typically present following trauma [30].

Despite the strengths of this study, there are also limitations. First, this study focused on the perceptions of women who had experienced complications with mesh implants and purposive sampling was used for recruitment. Many women who have had mesh implants have had positive experiences, with few or no difficulties, and this needs to be acknowledged. Second, the present study recruited women from an online support group and the Southeast of England whose experiences may be different from those who choose not to join such groups. Thus, future research should be aimed at recruiting a more diverse group based on socioeconomic, cultural and/or racial differences to reflect broader demographics within the population.

As an initial study on the impacts of vaginal mesh, this research gives an insight into the experiences of women with vaginal mesh complications. This study has highlighted the extensive impact that mesh has had on participants' relationships, in terms of both sexual activity and support given. Therefore, future research could explore the impact on partners of those with complications, giving valuable insight into the partners' experiences, how it impacts them and how they perceive their partners’ experiences. Further to this, aspects of the women’s experiences highlighted in this study could be investigated in greater depth, such as their feelings of an altered identity.

In conclusion, this study highlights how wide-reaching and life-changing vaginal mesh complications can be. It demonstrates the extensive impacts that vaginal mesh complications can have on women in terms of psychological distress, strained intimate relationships and lack of support from medical professionals. These results may help to raise awareness of the struggles of women experiencing incontinence and considering a mesh implant. Greater knowledge in this area is needed to raise awareness for both clinicians and patients when making medical decisions about mesh implants. This awareness will also benefit clinicians in providing much needed support for women experiencing complications with vaginal mesh, and will help to ensure that reported symptoms are taken seriously to prevent further damage.
